# Supplementary material for: Children with autism spectrum disorder and attention deficit hyperactivity disorder have evidence of sensory nerve degeneration
Source: PLoS One. 2026 Feb 23;21(2):e0342439. doi: 10.1371/journal.pone.0342439 (PMC12928439; doi:10.1371/journal.pone.0342439)
Supplement: Attachment — Submitted filename: Response to reviewers.pdf [file pone.0342439.s003.pdf]

22nd Oct 2025

Shivanand Kattimani
Academic Editor
PLOS ONE

PONE-D-25-44922
Children with Autism Spectrum Disorder and Attention Deficit Hyperactivity Disorder have Evidence of Sensory Nerve Degeneration

Dear Dr. Kattimani,

Thank you for providing us with the opportunity to revise our paper.
Please find attached a point-by-point response to the concerns raised. We hope that you find our responses satisfactory and that the manuscript is now acceptable for publication.

Kind Regards,

Rayaz A Malik, MD, PhD
Professor of Medicine,
Weill Cornell Medicine-Qatar,
Qatar Foundation,
Education City,
PO Box: 24144-Doha, Qatar

**Editorial comments**
Comment 1:<br>Please ensure that your manuscript meets PLOS ONE's style requirements, including those for file naming. The PLOS ONE style templates can be found at https://journals.plos.org/plosone/s/file?id=wjVg/PLOSOne_formatting_sample_main_body.pdf and https://journals.plos.org/plosone/s/file?id=ba62/PLOSOne_formatting_sample_title_authors_affiliations.pdf<br><br>Response 1:<br>Done.
Comment 2:<br>All PLOS journals now require all data underlying the findings described in their manuscript to be freely available to other researchers, either 1. In a public repository, 2. Within the manuscript itself, or 3. Uploaded as supplementary information.<br>This policy applies to all data except where public deposition would breach compliance with the protocol approved by your research ethics board. If your data cannot be made publicly available for ethical or legal reasons (e.g., public availability would compromise patient privacy), please explain your reasons on resubmission and your exemption request will be escalated for approval.<br><br>Response 2:<br>The data is now available as supplementary material in this paper, page 11.
Comment 3:<br>We note that the grant information you provided in the ‘Funding Information’ and ‘Financial Disclosure’ sections do not match.<br>When you resubmit, please ensure that you provide the correct grant numbers for the awards you received for your study in the ‘Funding Information’ section.<br><br>Response 3:<br>Thank you. This has been updated and there was no grant number for this award.
Comment 4:<br><br>Thank you for stating the following financial disclosure:<br>This study was funded by the Autism Research Institute<br><br>Please state what role the funders took in the study. If the funders had no role, please state: "The funders had no role in study design, data collection and analysis, decision to publish, or preparation of the manuscript." If this statement is not correct you must amend it as needed.

Weill Cornell Medicine

MANCHESTER 1824
The University of Manchester

Please include this amended Role of Funder statement in your cover letter; we will change the online submission form on your behalf.

Response 4:
This has been updated on page 11.

Comment 5:
Thank you for stating the following in your Competing Interests section:
None.
Please complete your Competing Interests on the online submission form to state any Competing Interests. If you have no competing interests, please state "The authors have declared that no competing interests exist.", as detailed online in our guide for authors at http://journals.plos.org/plosone/s/submit-now
This information should be included in your cover letter; we will change the online submission form on your behalf.

Response 5:
Thank you, we have now added this to the cover letter.

Comment 6:
Please amend your authorship list in your manuscript file to include author Mohammed A. Tolefat, Abdulla A. Alhothi.

Response 6:
Done.

Comment 7:
Please amend the manuscript submission data (via Edit Submission) to include author Mohammed Tolefat, Abdulla Alhothi.

Response 7:
Done.

Comment 8:
We note that there is identifying data in the Supporting Information file <19-000016_Initial Approval_03172020 (002).pdf>. Due to the inclusion of these potentially identifying data, we have removed this file from your file inventory. Prior to sharing human research participant data, authors should consult with an ethics committee to ensure data are shared in accordance with participant consent and all applicable local laws.

Data sharing should never compromise participant privacy. It is therefore not appropriate to publicly share personally identifiable data on human research participants. The following are examples of data that should not be shared:

-Name, initials, physical address

-Ages more specific than whole numbers
-Internet protocol (IP) address
-Specific dates (birth dates, death dates, examination dates, etc.)
-Contact information such as phone number or email address
-Location data
-ID numbers that seem specific (long numbers, include initials, titled “Hospital ID”) rather than random (small numbers in numerical order)

Data that are not directly identifying may also be inappropriate to share, as in combination they can become identifying. For example, data collected from a small group of participants, vulnerable populations, or private groups should not be shared if they involve indirect identifiers (such as sex, ethnicity, location, etc.) that may risk the identification of study participants.

Additional guidance on preparing raw data for publication can be found in our Data Policy (https://journals.plos.org/plosone/s/data-availability#loc-human-research-participant-data-and-other-sensitive-data) and in the following article: http://www.bmj.com/content/340/bmj.c181.long.

Please remove or anonymize all personal information (<specific identifying information in file to be removed>), ensure that the data shared are in accordance with participant consent, and re-upload a fully anonymized data set. Please note that spreadsheet columns with personal information must be removed and not hidden as all hidden columns will appear in the published file.

Response 8:
This was the ethical approval letter as requested during the submission, which includes a list of documents reviewed.
No identifier information are usually included in an IRB approval letter or even submitted to the ethics committee.
Additionnaly raw data has no identifying information.

Comment 9:
Please include captions for your Supporting Information files at the end of your manuscript, and update any in-text citations to match accordingly. Please see our Supporting Information guidelines for more information: http://journals.plos.org/plosone/s/supporting-information.

Response 9:
Added on page 11.

Reviewer 1

Comment 1:

This is an interesting and potentially important pilot study investigating corneal confocal microscopy (CCM) measures of sensory nerve degeneration in children with ASD, with and without ADHD. The topic is novel, as few studies have examined peripheral neurodegeneration in pediatric neurodevelopmental conditions. The manuscript is generally well written and clearly structured, but there are several major and minor issues that should be addressed to strengthen the scientific rigor and clarity.

Response 1:

Thank you for the kind feedback. We have addressed all the comments raised below with a point-by-point response and changes in track-changes.

Comment 2:

Major Comments

The study included 21 children with ASD+ADHD, 6 with ASD only, and 15 healthy controls. The very small sample size, especially for the ASD-only group, raises concerns about statistical power and generalizability. The authors should acknowledge this limitation more explicitly and discuss the implications for interpretation. A priori or post hoc power analysis would be helpful to clarify what effect sizes could reasonably be detected with the available sample, and to aid in the interpretation of null findings.

Response 2:

Thank you, we agree the small sample size is a limitation of our study and may limit the generalizability of our findings. As suggested, we have undertaken a post-hoc sample size calculation based on the CNFL difference between ASD+ADHD and ASD-ADHD groups, with α=0.05 and β=80% while accounting fot 10% loss of follow-up. This reveals that we require n=51 per group to rearch statistical significance. We have aknowledged this as a limitation in the discussion, page 10.

Below are the details for the sample size calculation:

CNFL									
α	$Z_{1-\alpha/2}$	Power	$Z_{1-\beta}$	$\mu_{intervention}$	$\mu_{standard}$	$\delta_{standard}$	Effect size	Sample size per arm	10% loss to follow up
0.05	1.96	80%	0.842	17.6	20	4.12	-0.5825243	46	51

Comment 3:

The design lacks a group of children with ADHD only, which limits the ability to disentangle whether the observed differences are specific to ASD or also present in ADHD. This gap should be acknowledged not only in the limitations but also in the rationale for study design, with an explanation of why the study remains valuable as an exploratory investigation. Where possible, the authors may cite existing literature on sensory nerve changes in ADHD.

Response 3:

We acknowledge that the absence of an ADHD-only group limits the ability to determine whether the observed differences are specific to ASD or also present in ADHD. We have now acknowledged this in the introduction (page 3-5) and in the limitations in the discussion (page 10).

Comment 4:

Beyond age and sex, it is unclear whether other potential confounding factors were considered, such as medication use, comorbid conditions, sleep problems, or ophthalmological disorders. These factors could plausibly affect corneal confocal microscopy (CCM) outcomes and should be addressed either in the methods or the discussion.

Response 4:

We agree that the factors such as medication use, comorbidities, sleep problems, and ophthalmological disorders could influence CCM outcomes. Unfortunately, this information was not available for our cohort, and we have now acknowledged this as a limitation in the discussion (page 10).

Comment 5:

Statistical analysis details

The manuscript requires more detail on the statistical procedures. Specifically:

(1)Were normality and homogeneity of variances checked?

(2)Given the small sample sizes, what statistical tests were applied (e.g., t-test, Mann–Whitney, ANOVA)?

(3)Was any correction for multiple comparisons (e.g., Bonferroni or FDR) performed?

(4)Were effect sizes or confidence intervals reported?

These details are essential for evaluating the reliability of the results.

Response 5:

We have provided all the above details on page 7 in the statistical analysis section and clarified that Bonferroni adjustment was used.

Comment 6:

Additional methodological detail on CCM protocols is warranted. For example, how many images were acquired per participant, and what criteria were used to select images (random vs. best quality)? Were images analyzed by a single rater or multiple raters, and was intra-rater or inter-rater reliability assessed? These factors are important for assessing the validity and reproducibility of the findings.

Response 6:

We agree and have now added these details in the CCM methods section, page 7.

Comment 7:

While statistically significant reductions in CNBD and CNFL were reported, the clinical meaning of these changes is not well elaborated. The authors should discuss whether the degree of

reduction is likely to have functional consequences, and how CCM could potentially be translated into clinical practice for ASD/ADHD populations.<br><br><u>Response 7:</u><br>Thank you.<br>We agree the clinical significance of the statistically significant reduction in CNBD and CNFL warrants further discussion. We have accordingly elaborated on the clinical implications of the changes in CCM in the discussion, page 9.
<u>Comment 8:</u><br>The lack of correlation between ASD severity scores and nerve measures is an important negative finding. This deserves more in-depth discussion. Does this suggest that sensory nerve degeneration is independent of behavioral symptom severity, or could it be related to limitations of the DSM-5-based severity scale?<br><br><u>Response 8:</u><br>Thank you for highlighting this important point, we have now expanded on this in the discussion section, page 9-10.
<u>Comment 9:</u><br>The conclusion states that ADHD does not confer additional neurodegeneration in children with ASD. Given the small subgroup sizes (n=6 ASD-only, n=21 ASD+ADHD), such conclusions may be premature. The authors should temper their interpretation and frame the results as preliminary.<br><br><u>Response 9:</u><br>We have updated the conclusion to reflect a more cautious interpretation, page 10.
<u>Comment 10:</u><br>Abstract: The abstract is clear but should briefly state the small sample size and the absence of an ADHD-only group to frame the findings appropriately.<br><br><u>Response 10:</u><br>Done, page 2.
<u>Comment 11:</u><br>Typographical correction in group labels: In the tables, the group name “ASD-AHDH” appears, which seems to be a typographical error for “ASD-ADHD.” Please correct this and ensure consistent labeling of groups across all tables, figures, and text.<br><br><u>Response 11:</u><br>Thank you. Done.
<u>Comment 12:</u><br>Methods: The description of statistical tests should be expanded (e.g., correction for multiple comparisons? effect size reporting?).

<u>Response 12:</u><br>Done, page 7.
<u>Comment 13:</u><br>Consistency of group labeling: The group names are not entirely consistent between the tables and figures (e.g., “ASD only” vs. “Autism,” “ASD+ADHD” vs. “Comorbid group”). The authors should standardize terminology across the text, tables, and figures to avoid confusion for readers. Using uniform labels (e.g., “ASD only,” “ASD+ADHD,” “Controls”) throughout the manuscript is strongly recommended.<br>In the tables, the group name “ASD-AHDH” appears, which seems to be a typographical error for “ASD-ADHD.” Please correct this and ensure consistent labeling of groups across all tables, figures, and text.<br><br><u>Response 13:</u><br>Thank you, we have revised the manuscript and unified the lables thoughout the manuscript.
<u>Comment 14:</u><br>Discussion: The discussion could better integrate existing literature on peripheral nerve abnormalities in ASD (e.g., Chien et al., 2020, Neurology) and ADHD-related sensory processing studies.<br><br><u>Response 14:</u><br>Thank you for the recommendation, we have expanded the discussion and included this reference, page 9.
<u>Comment 15:</u><br>References: Ensure all references are updated and formatted consistently.<br><br><u>Response 15:</u><br>Reformatted for PlosONE.
**Reviewer 2**
<u>Comment 1:</u><br>Introduction<br>- Lines 76–78: The phrasing is awkward, as ASD and ADHD are “bundled together” in a way that blurs their distinct definitions. This opening sentence should be rewritten to introduce each condition clearly before addressing their overlap.<br><br><u>Response 1:</u><br>Thank you, we have now defined both conditions in the introduction, page 3.
<u>Comment 2:</u><br>Introduction<br>- Lines 80–82: The statement about atypical sensory features being a “core diagnostic criterion in DSM-5” is not entirely correct. While sensory peculiarities are explicitly

acknowledged in the DSM-5 criteria, they were already well established in diagnostic tools such as the ADI-R and other scales. I agree that sensory issues are central in ASD, but the nuance is missed here: they are often underestimated (e.g., agitation misattributed to ADHD rather than sensory seeking) or overestimated (any sensory atypicality being interpreted as ASD). This complexity should be better reflected.<br><br><u>Response 2:</u><br>Thank you.<br>We agree that although DSM-5 incorporate both hyper- and hypo-activity as part of the diagnostic criteria, it doesn't treat atypical sensory features as an independent core diagnostic criterion of ASD.<br>We have now elaborated on this complex relationship in the introduction, page 4.
<u>Comment 3:</u><br>Introduction<br>- Line 84: Rather than describing individuals as hyposensitive to subjective pain intensity, it would be more accurate to state that they show particularities in pain perception, as both hyper- and hyposensitivity are documented.<br><br><u>Response 3:</u><br>Thank you, we have revised this in the introduction to indicate that people with ASD show particularities in pain perception, page 3,4.
<u>Comment 4:</u><br>Introduction<br>- Overall: The introduction is underdeveloped. It introduces non-consensual concepts (e.g., peripheral sensory nerve involvement) but does not defend them sufficiently. The innovative potential of these hypotheses needs to be articulated more clearly in relation to existing research that remains predominantly brain-centric. This contextualization would help readers understand why this line of inquiry matters.<br><br><u>Response 4:</u><br>Thank you, we have revised this section to emphasize on the importance of peripheral sensory involvement, page 5.
<u>Comment 5:</u><br>Introduction<br>- Lines 105–106: The manuscript lacks a clear hypothesis. Presenting corneal confocal microscopy (CCM) results in isolation risks being purely descriptive. The authors should articulate how detecting corneal alterations could improve understanding of patient experience, symptoms, or clinical care.

Response 5:<br>We have now rephrased the aim of the study, page 5.
Comment 6:<br>Methods<br>- The description of participants should clarify whether the ASD group included children with intellectual disability, as this is highly relevant to interpretation.<br>- Greater transparency is needed about recruitment strategy, representativeness, and diagnostic validation.<br><br>Response 6:<br>We have now clarified that the study included participants with mild-to-moderate ASD to enable CCM examination as it was not practical or possible in the non-verbal individuals with severe ASD.
Comment 7:<br>Results<br>- The small sample sizes (ASD without ADHD: n=6) limit statistical validity. Reporting a non-significant p=0.08 as evidence of no difference between groups is misleading. With such underpowered comparisons, absence of significance cannot be taken as equivalence.<br><br>Response 7:<br>We agree and have acknowledged this as a limitation in the discussion, page 11.
Comment 8:<br>General Assessment<br>The study proposes a potentially innovative shift from brain-centric to peripheral mechanisms in ASD/ADHD, but the introduction is too thin, the hypothesis is not clearly posed, and the interpretation is overstated given the sample size and reliance on prior self-citations. The paper would be strengthened by:<br>1. Rewriting the introduction for clarity and nuance (particularly around diagnostic criteria and sensory features).<br>2. Explicitly stating a testable hypothesis with clinical or mechanistic relevance.<br>3. Tempering claims where evidence is preliminary.<br>4. Expanding on the rationale for corneal measures—what do they add to patient understanding or care?<br><br>Response 8:<br>We are grateful for the reviewer's insightful comments. In response, we have refined the introduction to clarify the study's aim and have expanded the discussion to acknowledge the sample size limitation (page 9) and the clinical significance of using CCM in this context (page 11).

Reviewer 3
Comment 1:<br>The authors are correct to highlight the fact that the changes to diagnostic categorisation of autism in the DSM-5 in 2013 resulted in a new separate criterion relating to atypical/unusual responses to sensory stimuli. Furthermore, prior to DSM-5, it was not possible to diagnose autism if ADHD were indicated. With advances in the literature, the common co-occurring nature of these two conditions suggests clinical research should address both presentations in single diagnostic groups and those with multiple diagnoses. This pilot study might contribute to knowledge in this field.<br>The authors need to be clear from the outset that this is a pilot study.<br><br>Response 1:<br>Thank you for the kind feedback. We have stated in the title, methods and discussion that this is a pilot study.
Comment 2:<br>The abstract requires greater clarity about the aims of the study rather than simply what was done.<br><br>Response 2:<br>Done, page 2.
Comment 3:<br>Terminology is unclear, i.e., when authors refer to neurodegeneration, do they mean loss of functioning/deterioration or ophthalmologic abnormality? The former would lead me to assume the pilot study aims to review participants over time rather than comparing clinical and non-clinical groups. Cf p2 lines 47 and 56 and p3 line 96.<br><br>Response 3:<br>We have replaced the term neurodegeneration with nerve loss throughout the manuscript as we did not undertake any functional assessment such as corneal sensation.
Comment 4:<br>Methodology<br>The current study description is not adequate to understand the processes or participant selection and hence to replicate the study.<br><br>Response 4:<br>We have expanded the methods (page 5-7).
Comment 5:<br>Methodology<br>Please capitalise the names of the recruiting centres and explain their remit/role.<br><br>Response 5:

Done, and both sites were referred eligible participants for research, page 5.
<u>Comment 6:</u><br>Methodology<br>Furthermore, how were the healthy controls selected?<br><br><u>Response 6:</u><br>They were selected from a study which recruited healthy controls without any chronic conditions and who met the exclusion criteria for CCM, page 5.
<u>Comment 7:</u><br>Methodology<br>How many families were approached before the final sample was selected? Did anyone drop out?'<br><br><u>Response 7:</u><br>The family was approached for all indivduals recruited. However for three families who agreed, CCM was not possible as the children could not cooperate adequately (the results have been updated on page 8 to reflect this.
<u>Comment 8:</u><br>Methodology<br>The authors have not explained participant characteristics, including whether the children had an existing diagnosis and, if so, how this was ratified, such as brief assessment, questionnaires or tasks. For instance, in lines 80-81, the authors state 'there is an increasing recognition of abnormal sensory responses in ASD …which relate to quantitative autism traits… such that it now constitutes a core diagnostic [criterion] in DSM-5…'<br><br><u>Response 8:</u><br>Unfortunately, we did not have acces to additional findings on examination in the clinic- only the diagnosis from their medical records.
<u>Comment 9:</u><br>Methodology<br>There is no description of validation of the autism diagnoses, how children were identified or recruited, and the opportunity was lost to investigate any quantitative traits, such as those identified via completion of questionnaires and establishing whether the sensory differences criterion was met for all the autistic children and young people.<br><br><u>Response 9:</u><br>Thank you. The diagnosis of ASD and ADHD was made by senior clinicians specialising in the care of children with ASD and ADHD in the participants routine clinic visit and our study extracted data from the electronic medical records. As such, we did not have access to standardized diagnostic tools or questionnaires for sensory phenotyping. This has been clarified in the Methods section.

Comment 10:
Methodology
Symptom severity for both autism and ADHD require operational definitions.

Response 10:
Thank you. The severity of ASD was based on the DSM-5, according to the levels of support needed in social communication and restricted/repetitive behaviors, rated from Level 1 (requiring support) to Level 3 (requiring very substantial support). This has been clarified in the methods section.

Comment 11:
Methodology
The pilot study was small, with little detail about the range of participants, including cognitive ability.

Response 11:
We have provided further details in the methods page 5. We included only children with mild-to-moderate ASD to allow for CCM examination.

Comment 12:
Methodology
Whilst age differences were not statistically significant, the non-autistic children were in general older than the autistic children. A table summarising the age ranges for all groups would be helpful.

Response 12:
We have summarized the age of each group in table 1, page 8.

Comment 13:
Diagnostic criteria
It is not clear from this section whether the children were assessed and diagnosed as part of this study or whether they were recruited via a clinic where they already had received a diagnosis.

Response 13:
We have emphasized that these children were diagnosed already and diagnosis was made as part of clinical care, page 6.

Comment 14:
Diagnostic criteria
2. It is not noted how they were assessed, and which, if any tools supported the assessment and diagnostic process. Did the researchers have access to the clinical diagnostic assessment details?

Response 14:

Weill Cornell Medicine

MANCHESTER 1824
The University of Manchester

This was done as part of clinical care and data were accessed via the participant's health records. This was not done as part of the research assessment. This has been clarified in the methods, page 6.
<u>Comment 15:</u><br>Corneal Confocal Microscopy<br>1. Who conducted this?<br>2. At the end of the paragraph, it is noted that the investigator was blind to the images but does not say who conducted the procedure.<br><br><u>Response 15:</u><br>The image acquisition, selction and analysis was done by HG (as stated in the methods section).
<u>Comment 16:</u><br>Discussion<br>P7 line 196 - This is not my field of expertise, but do the results demonstrate 'loss' or difference/pathology between groups?<br><br><u>Response 16:</u><br>The results demonstrate corneal nerve loss as evidenced by multiple studies in other peripheral and central neurodegenerative conditions.
<u>Comment 17:</u><br>Discussion<br>P8 line 202 – refer to ELENA cohort earlier in the paper or explain it here.<br><br><u>Response 17:</u><br>Defined and cited in page 3.
<u>Comment 18:</u><br>Discussion<br>The authors only identify two limitations, including the importance of objective measures of severity – this, in my mind is significant. I would argue that the findings are limited, and it is unclear what the next steps might be or implications of future research or indeed clinical impact.<br><br><u>Response 18:</u><br>We have acknowledged additional limitations, including the exploratory nature of the findings, and the lack of ADHD-alone group, page 11.
<u>Comment 19:</u><br><br>Typos<br>1. P3 line 82 criteria should read criterion<br>2. P5, line 121, capitalise the full name of the manual and ensure that throughout the manuscript it is written correctly

3. P6 line 163 correlate<br>4. P7 Table 1 typos ADHD rather than AHDH<br>5. P8 ‘additonal’ should read additional?<br><br><u>Response 19:</u><br>Thank you, all corrected.
