# Supplementary material for: Children with autism spectrum disorder and attention deficit hyperactivity disorder have evidence of sensory nerve degeneration
Source: PLoS One. 2026 Feb 23;21(2):e0342439. doi: 10.1371/journal.pone.0342439 (PMC12928439; doi:10.1371/journal.pone.0342439)
Supplement: Attachment — Submitted filename: Response_to_reviewers_auresp_2.pdf [file pone.0342439.s004.pdf]

Weill Cornell Medicine

MANCHESTER 1824
The University of Manchester

19th November 2025

Shivanand Kattimani
Academic Editor
PLOS ONE

PONE-D-25-44922R1
Children with Autism Spectrum Disorder and Attention Deficit Hyperactivity Disorder have Evidence of Sensory Nerve Degeneration

Dear Dr. Kattimani,

Thank you for providing us with the opportunity to address further concerns by the reviewers and to revise our paper.
Please find attached a point-by-point response to the concerns raised and hope that you find our responses satisfactory and that the manuscript is now acceptable for publication.

Kind Regards,

Rayaz A Malik, MD, PhD
Professor of Medicine,
Weill Cornell Medicine-Qatar,
Qatar Foundation,
Education City,
PO Box: 24144-Doha, Qatar

Reviewer 1
Comment 1:<br>The revision effectively clarifies methodology, improves the discussion's conceptual integration, and adopts a balanced interpretation of results. Minor linguistic polishing and the addition of a brief statement on the clinical relevance of sensory and motor assessments in future screening would enhance the paper's translational impact.<br>Below are some minor suggestions.<br>Abstract and Introduction: The introduction is concise and sets up the rationale well. However, the research objectives would benefit from a brief mention that this is a preliminary exploratory study aiming to inform future mechanistic investigations and clinical screening strategies.<br><br>Response 1:<br>Thank you, the abstract has been revised to state this is an exploratory study (page 2, line 46) and includes a statement on how this study may inform on the utility of CCM as a clinical screening strategy in subjects with ASD and ADHD (page 2).
Comment 2:<br>Methods: The methodological transparency has improved, but minor editorial polishing is needed for sentence flow. Clarifying how "routine clinical care" diagnoses were confirmed (e.g., by pediatric neurologist or psychiatrist) would strengthen credibility.<br><br>Response 2:<br>Thank you, we have now stated that "ASD and ADHD were diagnosed by consultant pediatricians with a specialist interest in behavioral pediatrics, ADHD and autism spectrum disorder, according to the Diagnostic and Statistical Manual of Mental Disorders – version 5 (DSM-5) (page 5).
Comment 3:<br>Results: the presentation of the results could be strengthened by providing greater quantitative detail and statistical context to assist readers in interpreting the effect magnitude and reliability. Specifically:<br>(1) Group comparison values (e.g., mean ± SD, p-values, or effect sizes such as Cohen's d or η^2) should be explicitly reported in-text or summarized in a table. This will allow readers to gauge the robustness of the observed differences beyond descriptive trends.<br>(2) It would be valuable to indicate whether the observed corneal nerve loss differed significantly between the ASD and ASD+ADHD subgroups, given the study's stated aim of exploring comorbid ADHD effects. Even if the difference did not reach significance, reporting the directionality and confidence intervals would increase transparency.<br>(3) The authors may consider briefly stating which specific CCM parameters (e.g., corneal

nerve fiber density, length, branch density) contributed most strongly to the group differences.
(4) Including a visual representation (figure or boxplot) summarizing group distributions could substantially enhance clarity and reader engagement, particularly for an exploratory study with small sample sizes.

Response 3:

1. Thank you we have now revised the results section to include explicit quantitative details in addition to group means ± SD and p-values we also include effect size (using Cohen's d or eta-squared) where appropriate, methods page 7 and revised table 1, page 8.
2. We have now included a direct comparison between the ASD and ASD+ADHD subgroups in the results section, page 8. We have also reported the mean differences, 95% confidence intervals, and directionality of the effect to enhance transparency and align with the study's stated aim of exploring comorbid ADHD effects. This has been stated on page 8.
3. We have clarified in the results section which specific CCM parameters contributed most strongly to the group differences, page 8.
4. Figure 1. Represents the distribution of CNFD, CNBD and CNFL in scatter plots in all 3 groups.

Comment 4:
Discussion: The discussion could be further strengthened by adding a short clinical implication statement, for example:
"In future screening and follow-up protocols, it may be valuable to include comprehensive evaluations of motor coordination and visual–perceptual abilities, allowing for the early identification of subtle neurodevelopmental difficulties that could precede or co-occur with psychosocial challenges in children with ASD or ASD+ADHD."

Response 4:
Thank you. As suggested, we have added a statement outlining the potential clinical implications of our findings in the discussion, (page 11).

Comment 5:
Limitation and conclusion: The conclusion could end on a more forward-looking note, highlighting that future multimodal investigations combining corneal confocal microscopy with sensory and cognitive assessments could elucidate mechanisms of neurodevelopmental comorbidity.

Response 5:
Thank you for your valuable suggestion. We have revised our conclusion, (page 11).

**Reviewer 3**
<u>Comment 1:</u><br>I continue to believe the authors have not clarified enough the likely benefit of the findings of the current pilot or potential benefits if a larger study were conducted. Despite the revision of the abstract I am still unclear.<br><br><u>Response 1:</u><br>Thank you. We have revised the abstract to clearly articulate the potential benefits of our findings and the rationale for future larger studies (page 2).
<u>Comment 2:</u><br>lines 59 -60 ' Children with ASD+ADHD with and without ADHDASD-alone have evidence of sensory nerve<br>degeneration'. - typos<br><br><u>Response 2:</u><br>Thank you, corrected (page 2).
<u>Comment 3:</u><br>lines 86-88 'It is highly heterogeneous being varied in its presentation, co-occurring with conditions like intellectual disability, and is shaped by factors such as age, gender, race/ethnicity, and developmental stage[1]' - this reads as thought ID commonly co-occurs with autism, whereas it is more likely that autistic people do not have additional ID. mental health conditions might be more relevant to add here.<br><br><u>Response 3:</u><br>Thank you for this comment. We have now revised the sentence to 'It can vary in its presentation may be associated with anxiety and depression and shaped by age, gender, race/ethnicity, and developmental stage' (page 3).
<u>Comment 4:</u><br>Lines 95-98 'Most research has focused on brain centric mechanisms in both ASD and ADHD, however, there iss an increasing recognition of abnormal sensory responses in ASD [5-10] which relate to quantitative autism traits [7] such that it now constitutes one of the four sub-criteria under the restricted, repetitive behaviors domain a core diagnostic criteria in DSM-5 diagnostic criteria [11], but however this does not take into account the atypical sensory features of ASD' - this sentence should be rephrased and shortened/separated into two sentences and typos corrected.<br><br><u>Response 4:</u><br>Thank you. We have now revised this section. "Most research has focused on brain-centric mechanisms in ASD and ADHD. However, there is increasing recognition of abnormal sensory responses in ASD which relate to quantitative autism traits such that they constitute one of the

four sub-criteria under the restricted, repetitive behaviors domain in the Diagnostic and Statistical Manual of Mental Disorders, Fifth Edition **(**DSM-5)", (page 3).
<u>Comment 5:</u><br>'Subjects with ASD' - check for consistency in terminology - subjects, patients or participants<br><br><u>Response 5:</u><br>Thank you, we have now ensured consistent terminology, using "participants" throughout to refer to all study subjects.
<u>Comment 6:</u><br>'Eligible participants with ASD were referred' - is this the case? did paediatricians actually refer patients or did they provide information about the research?<br><br><u>Response 6:</u><br>Thank you, we have now clarified the process of diagnosis and assessment, (page 5).<br>ASD and ADHD were diagnosed by consultant pediatricians with a specialist interest in behavioral pediatrics, ADHD and ASD, according to the Diagnostic and Statistical Manual of Mental Disorders – version 5 (DSM-5), (page 5).
<u>Comment 7:</u><br>'healthy controls were selected from the general pediatrics clinicstudy' -- this is unclear to me. Do all children regularly visit paediatric clinics for review or evaluation or are they referred if there are concerns? this may be different across international systems.<br><br><u>Response 7:</u><br>We have now clarified how the healthy controls attending pediatric clinics were recruited, (page 5, line 118-121).,<br>"Healthy controls (HC) were children attending the pediatric clinic for follow up of acute conditions, without neurodevelopmental or chronic medical conditions, meeting the exclusion criteria for CCM".
<u>Comment 8:</u><br>'ASD was diagnosed as part of clinical care' - it still reads as though the children were assessed during the study - if they had already been assessed - i.e., some months or years earlier this should be clarified.<br><br><u>Response 8:</u><br>Thank you for this comment. We have now clarified that the ASD diagnosis made by specialists was extracted from the electronic medical records (EMR), (page 6).<br>We did not have access to standardized diagnostic tools or questionnaires for each participant as data on diagnosis was extracted from the electronic medical record.
<u>Comment 9:</u><br>'DSM5' to be written as DSM-5.

Response 9:<br>We have corrected this.
Comment 10:<br>Overall, I am uncertain of the conclusions being drawn, and ability to replicate this study, given the methodology.<br><br>Response 10:<br>We have now refined the results, discusison and conclusion to acknowledge the study's limitations, including small sample size and preliminary findings.<br><br>"From a clinical perspective, these findings highlight the potential value of incorporating comprehensive evaluations of motor coordination and visual–perceptual abilities alongside CCM, into future screening protocols to identify subtle neurodevelopmental difficulties that precede or co-occur with psychosocial challenges in children with ASD or ASD+ADHD", (Page 11).
