# Supplementary material for: Children with autism spectrum disorder and attention deficit hyperactivity disorder have evidence of sensory nerve degeneration
Source: PLoS One. 2026 Feb 23;21(2):e0342439. doi: 10.1371/journal.pone.0342439 (PMC12928439; doi:10.1371/journal.pone.0342439)
Supplement: Attachment — Submitted filename: Response_to_reviewers_auresp_3.pdf [file pone.0342439.s005.pdf]

21st Dec 2025

Shivanand Kattimani
Academic Editor
PLOS ONE

PONE-D-25-44922R2
Children with Autism Spectrum Disorder and Attention Deficit Hyperactivity Disorder have Evidence of Sensory Nerve Degeneration

Dear Dr. Kattimani,

Thank you for providing us with the opportunity to address further concerns by the reviewers. Please find attached a point-by-point response to the concerns raised and hope that you find our responses satisfactory and that the manuscript is now acceptable for publication.

Kind Regards,

Rayaz A Malik, MD, PhD
Professor of Medicine,
Weill Cornell Medicine-Qatar,
Qatar Foundation,
Education City,
PO Box: 24144-Doha, Qatar

**Reviewer 1**
Comment 1:<br>The revised version of the manuscript is much improved, with clearer presentation and a more focused interpretation. However, regarding the initial hypothesis that ASD+ADHD would show greater corneal nerve degeneration than ASD alone, the Discussion does not yet fully explain why this expectation was not supported. Beyond noting the small sample size and the absence of an ADHD-only comparison group, the authors could strengthen the Discussion by briefly considering several additional factors that may account for the non-significant findings:<br><br>Response 1:<br>Thank you for your valuable suggestions. We have now elaborated on the below points in the discussion.
Comment 2:<br>Age differences between groups and developmental variation in corneal nerve parameters that could influence group comparisons;<br><br>The potential influence of ADHD neurobiology or stimulant medications, which may modulate peripheral nerve function and confound direct comparisons;<br><br>Heterogeneity in sensory-processing profiles across ASD and ADHD, which may result in distinct peripheral nerve patterns not captured by group-level analyses;<br><br>Response 2:<br>We agree that developmental changes in corneal nerve morphology may influence group comparisons, we have now discussed that in page 9.<br>We also acknowledge differences in sensory-processing profiles between ASD and ADHD and the potential effect of pharmacological treatment effect on nerves in the discussion, page 9.
Comment 3:<br>Interpretation of effect sizes and confidence intervals, to clarify whether the lack of significant differences reflects a true absence of group effects or insufficient statistical power.<br><br>Response 3:<br>We have expanded the interpretation of the effect size in the discussion, page 11.
Comment 4:<br>The author may incorporate these points would provide a more comprehensive explanation for why the initial hypothesis was not confirmed and would help contextualize the findings within broader neurodevelopmental and sensory-processing frameworks.<br><br>Response 4:<br>Done.
**Reviewer 3**

<u>Comment 1:</u><br>I am satisfied that the authors have addressed the core concerns. I only have two minor typos to highlight:<br><br><u>Response 1:</u><br>Thank you for your kind feedback.
<u>Comment 2:</u><br>p3 . It can vary in its presentation may be associated with anxiety and depression and shaped by age, gender,<br>race/ethnicity, and developmental stage [1]. COMMA MISSING<br>P4 Parkinson disease - Parkinson's disease<br><br><u>Response 2:</u><br>Thank you, done on page 3 and 4.
