# Supplementary material for: Children with autism spectrum disorder and attention deficit hyperactivity disorder have evidence of sensory nerve degeneration
Source: PLoS One. 2026 Feb 23;21(2):e0342439. doi: 10.1371/journal.pone.0342439 (PMC12928439; doi:10.1371/journal.pone.0342439)
Supplement: Attachment — Submitted filename: Response_to_reviewers_auresp_4.pdf [file pone.0342439.s006.pdf]

20th Jan 2026

Shivanand Kattimani
Academic Editor
PLOS ONE

PONE-D-25-44922R3
Children with Autism Spectrum Disorder and Attention Deficit Hyperactivity Disorder have Evidence of Sensory Nerve Degeneration

Dear Dr. Kattimani,

Thank you for providing us with the opportunity to address further concerns by reviewer 3. Please find attached a point-by-point response to the concerns raised and hope that you find our responses satisfactory and that the manuscript is now acceptable for publication.

Kind Regards,

Rayaz A Malik, MD, PhD
Professor of Medicine,
Weill Cornell Medicine-Qatar,
Qatar Foundation,
Education City,
PO Box: 24144-Doha, Qatar

Reviewer 1
<u>Comment 1:</u><br>I am satisfied with the revised manuscript. Thank you for the authors' efforts, and I look forward to seeing the paper published.<br><br><u>Response 1:</u><br>Thank you.
**Reviewer 3**
<u>Comment 1:</u><br>Thanks you for addressing the comments. I have two minor points to add with respect to your responses to Reviewer 1's comments.<br>Abstract needs to acknowledge the groups studied, particularly since an ADHD group alone was not included: 'a clinical screening strategy for neurodegneration in ASD and ADHD' should this read and ASD-ADHD combined?<br><br><u>Response 1:</u><br>Thank you, we have revised the abstract to explicitly define the study groups, page 2.
<u>Comment 2:</u><br>Page 10 outlines the limitations of the exploratory study including group size. Since the groups differed with respect to age, should this also be added?<br><br><u>Response 2:</u><br>Thank you, we have revised the limitations to acknowledge the potential impact of age-related effects on corneal nerve measures, page 10.
